# Macrophage Trafficking as Key Mediator of Adenine-Induced Kidney Injury

**DOI:** 10.1155/2014/291024

**Published:** 2014-07-16

**Authors:** Matheus Correa-Costa, Tárcio Teodoro Braga, Raphael José Ferreira Felizardo, Vinícius Andrade-Oliveira, Katia Regina Perez, Iolanda Midea Cuccovia, Meire Ioshie Hiyane, João Santana da Silva, Niels Olsen Saraiva Câmara

**Affiliations:** ^1^Laboratory of Transplantation Immunobiology, Department of Immunology, University of São Paulo, Institute of Biomedical Sciences, 05508-900 São Paulo, SP, Brazil; ^2^Laboratory of Clinical and Experimental Immunology, Nephrology Division, Federal University of São Paulo, 04023-900 São Paulo, SP, Brazil; ^3^Department of Biophysics, Federal University of São Paulo, 04023-062 São Paulo, SP, Brazil; ^4^Institute of Chemistry, Department of Biochemistry, University of São Paulo, 05508-000 São Paulo, SP, Brazil; ^5^Department of Biochemistry and Immunology, School of Medicine of Ribeirão Preto, University of São Paulo, 14049-900 Ribeirão Preto, SP, Brazil

## Abstract

Macrophages play a special role in the onset of several diseases, including acute and chronic kidney injuries. In this sense, tubule interstitial nephritis (TIN) represents an underestimated insult, which can be triggered by different stimuli and, in the absence of a proper regulation, can lead to fibrosis deposition. Based on this perception, we evaluated the participation of macrophage recruitment in the development of TIN. Initially, we provided adenine-enriched food to WT and searched for macrophage presence and action in the kidney. Also, a group of animals were depleted of macrophages with the clodronate liposome while receiving adenine-enriched diet. We collected blood and renal tissue from these animals and renal function, inflammation, and fibrosis were evaluated. We observed higher expression of chemokines in the kidneys of adenine-fed mice and a substantial protection when macrophages were depleted. Then, we specifically investigated the role of some key chemokines, CCR5 and CCL3, in this TIN experimental model. Interestingly, CCR5 KO and CCL3 KO animals showed less renal dysfunction and a decreased proinflammatory profile. Furthermore, in those animals, there was less profibrotic signaling. In conclusion, we can suggest that macrophage infiltration is important for the onset of renal injury in the adenine-induced TIN.

## 1. Introduction

The development of chronic kidney disease is a result of a series of actions, among which are inflammation and interstitial fibrosis, with consequent vascular and tubular atrophy. In this scenario, macrophages seem to be involved in all these events, although its specific contribution has not been fully elucidated [1]. Although there are some resident macrophages in the kidney, during the development of renal damage recruitment of more of this cell lineage to the organ occurs. This trafficking is mediated by molecules called chemokines, which direct the movement of circulating leukocytes to the sites of inflammation [2].

More than 50 chemokines have been described, including CCL2, CCL3, and CCL5, and this “recruitment process” appears to contribute effectively to the progression from an acute insult to a chronic renal injury [3]. These chemokines act on specific receptors; for example, the CC-chemokine receptor 5 (CCR5), a member of CC-chemokine receptor family, is a seven-transmembrane G protein-coupled receptor (GPCR) and is responsible for trafficking and effector functions of macrophages, T cells, dendritic cells, and NK cells [4].

Recently, a study demonstrated that the macrophage population mediates kidney injury in the obstructive model of chronic kidney disease [5], but their role in other models of renal insults still needs to be better understood. As these cells are important mediators of the inflammatory process elements, we decided to investigate their role in an experimental model of tubule-interstitial nephritis (TIN). An intense inflammatory process in the tubule interstitial compartment characterizes this disease and the persistence of the stimulus triggering the insult leads to a chronic phase of the disease and subsequent formation of fibrosis [6]. Moreover, we have previously observed that, in the adenine model of renal injury, the lack of some of the innate immune compounds activation leads to a protective profile, which makes us speculate that the macrophages population might be involved in this disease. Then, we hypothesized that lack of macrophage recruitment and infiltration into the injured tissue attenuates renal dysfunction, inflammation, and, ultimately, fibrosis.

## 2. Methods

### 2.1. Animals and Reagents

CCR5 and CCL3 knockout mice were purchased from School of Medicine of Ribeirão Preto, University of Sao Paulo. C57BL/6 (WT) mice were obtained from our Isogenic Breeding Unit (Immunology Department, Biomedical Science Institute, University of Sao Paulo). The knockout mice have the C57BL/6 genetic background and were backcrossed for 20 generations. All animals were used at 8–12 wk of age. Animals were fed with 0.25% adenine-enriched food (Rhoster, Aracoiaba da Serra, Brazil) for 10 days and were sacrificed thereafter. Control animals received standard food for the same period. All experimental procedures were done in accordance with ethical statements approved by the Institutional Ethical Committee of the University of Sao Paulo. The Ethical Committee approved the study after detailed analysis (document number 012/2010).

### 2.2. Biochemical Parameter Analyses

Blood was collected for serum creatinine measurements. All samples were analyzed by colorimetric assays using commercially purchased kit (Creatinine Kit, Labtest, Minas Gerais, Brazil).

### 2.3. Histomorphometric Analyses

Formaldehyde-fixed paraffin sections of the kidneys were stained with hematoxylin-eosin and picrosirius for evaluation of cellular infiltration, tubular dilation, and fibrosis deposition. Renal histomorphometric analyses were made by two “blinded” renal histologists. Tubular area was assessed by quantification of tubular spaces, and results are expressed as pixels. Picrosirius stained sections were analyzed by an Olympus BX50 microscope with an Olympus camera attached (USA). Manual shots were taken of the cortex, magnified 40x, and observed under polarized light. Photos of at least 5 different fields in each slide were taken, and structures such as the glomeruli, subcapsular cortex, large vessels, and medulla were excluded. The pictures were digitalized (HP Scanjet 2400) and then the interstitial volume of collagen in the cortex compared to the overall cortex area was quantified by morphometry. For the morphometric analysis, the Image Processing and Analysis in Java, Image J, software was used. The result of the analysis is represented by percentage and refers to the proportion of the interstitial volume of collagen in the cortex to the total cortical interstitial volume, and then the arithmetic mean of the analyzed fields was calculated for each slide. The IHC for CD11b (Abcam, USA) were performed following the instructions of the DAKO Envision technique.

### 2.4. Gene Profiles

Kidney samples were snap-frozen in liquid nitrogen. Total RNA was isolated from kidney tissue using the TRIzol Reagent (Invitrogen, Carlsbad, USA) and protocol according to Invitrogen. RNA concentrations were determined by spectrophotometer readings at absorbance 260 nm. First-strand cDNAs were synthesized using the MML-V reverse transcriptase (Promega, Madison, USA). RT-PCR was performed using the Taqman real-time PCR assay (Applied Biosystem, USA) for the following molecules: HPRT (Mm00446968_m1), TNF-*α* (Mm00443258_m1), IL-6 (Mm004461690_m1), KIM-1 (Mm00801778_m1), XDH (Mm00442110_m1), CCR5 (Mm01963251_s1), Arginase-1 (Mm01190441_g1), iNOS (Mm00440485_m1), CD68 (Mm03047340_m1), E-cadherin (Mm01247357_m1), and vimentin (Mm01333430_m1). Cycling conditions were as follows: 10 minutes at 95°C followed by 45 cycles at 20 seconds each at 95°C, 20 seconds at 58°C, and 20 seconds at 72°C. Analysis used Sequence Detection Software 1.9 (SDS). mRNA expression was normalized to HPRT expression.

### 2.5. Western Blot Analysis

Kidney cells were lysed in RIPA buffer, run on a 10% SDS-polyacrylamide electrophoresis gel, and transferred onto a nitrocellulose membrane (Hybond C Extra, Amersham Biosciences, Little Chalfont, USA). Membranes were incubated with primary rabbit anti-mouse phospho-IKK*α*/*β* (Cell Signaling #2697, MA, USA) antibody, using manufacturer-recommended dilutions, followed by a peroxidase-conjugated mouse anti-rabbit IgG antibody (Sigma, St. Louis, USA). HRP activity was detected using enhanced chemiluminescence. The membrane was stripped and probed with mouse primary anti-*β*-actin antibody (Sigma, St. Louis, USA) to confirm and estimate the loading and the transfer. We used the software GeneSnap (Syngene, USA) and Gene Tools (Syngene, USA) to analyze the bands.

### 2.6. Bio-Plex

Kidney cells were lysed in RIPA buffer with protease inhibitor. A Bio-Plex mouse Plex cytokine assay kit (Bio-Rad Laboratories, Inc., Hercules, CA, USA) was used to test samples for the presence of 15 cytokines. The assay was read on the Bio-Plex suspension array system, and the data were analyzed using Bio-Plex Manager software version 4.0. Standard curves ranged from 32,000 to 1.95 pg/mL.

### 2.7. Flow Cytometry

Macrophages infiltrating cells were analyzed by multicolor flow cytometry. The monoclonal antibody used was CD11b PE (purchased from BD Biosciences, Franklin Lakes, NJ, USA). Samples were acquired on a FACSCanto, using FACSDiva software (BD Biosciences), and then were analyzed with FlowJo software (Tree Star, Ashland, OR, USA). Fluorescence voltages were determined using matched unstained cells. Two hundred thousand events were acquired in a live mononuclear gate.

### 2.8. Liposome Preparation and Macrophage Depletion

Clodronate (Bonefos, Schering, São Paulo, Brazil) was entrapped in liposomes by ether injection as described as follows. Typically, 0.5 mL  of an ether solution containing 50 mg phosphatidylcholine and 8 mg cholesterol was injected (0.2 mL/minute) into 5 mL of a 50 mmol/L clodronate aqueous solution maintained at 42°C. The liposome suspension was centrifuged at 22,800 g for 30 min (Hitachi HimacCR20B2 Centrifuge, Hitachi, Troy, MI, USA) at 25°C. The liposome-containing pellet was washed twice by centrifugation under the same conditions in NaCl solution 0.9% (w/v). The final pellet was resuspended in 2 mL of saline solution. Typically, the final phosphatidylcholine and clodronate concentrations in the liposomes were 10 mmol/L and 0.5 mmol/L, respectively. The yield of entrapped clodronate was about 1% of the initial quantity added. Mice were injected intraperitoneally (IP) with 200 *μ*L of liposome preparation (6 *μ*g of clodronate) at days 1, 4, and 7 of adenine food administration.

### 2.9. Statistics

The data were described as mean ± S.E.M. Differences among groups were compared using ANOVA (with Tukey post-test) and Student* t*-test. Significant differences were regarded as *P* < 0.05. All statistical analyses were performed with the aid of GraphPad PRISM.

## 3. Results

### 3.1. Macrophage Recruitment Is Enhanced during Experimental TIN

**Figure 1:**
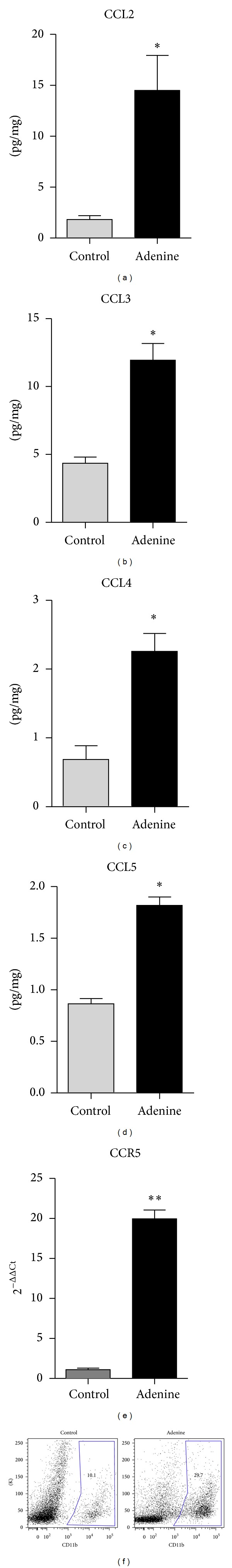
Adenine-enriched food increases the expression of chemokines and macrophages in renal tissue. Protein expression of CCL2 (a), CCL3 (b), CCL4 (c), and CCL5 (d) was measured in kidney lysates of animals that received standard food or adenine-enriched food. Panel (e) shows the gene expression of CCR5 in the same animals. In panel (f) the dot plot of macrophage infiltration is shown, observed by the CD11b marker. *P < 0.05 versus control and **P < 0.01 versus control.

In order to evaluate the role of macrophage trafficking during experimental TIN, we checked the protein expression of macrophages related chemokines after providing adenine-enriched chow to WT animals. As observed in Figures 1(a)–1(d), mice that received adenine food show a striking elevation in CCL2, CCL3, CCL4, and CCL5. All these molecules, to some extent, are responsible for macrophages recruitment. Moreover, there is an upregulation of CCR5 expression in the renal tissue of adenine-fed mice (Figure 1(e)). To confirm the hypothesis that all these molecules are important for cell recruitment, we evaluated the presence of infiltrating macrophages in renal tissue after excessive adenine intake. As seen in Figure 1(f), during TIN there is a marked increase of these cells in the renal tissue. Together, these data support the idea that macrophages take part of TIN and that during the development of this disease there is an increase in their recruitment signaling and trafficking.

### 3.2. Macrophage Depletion Protects from Adenine-Induced TIN

**Figure 2:**
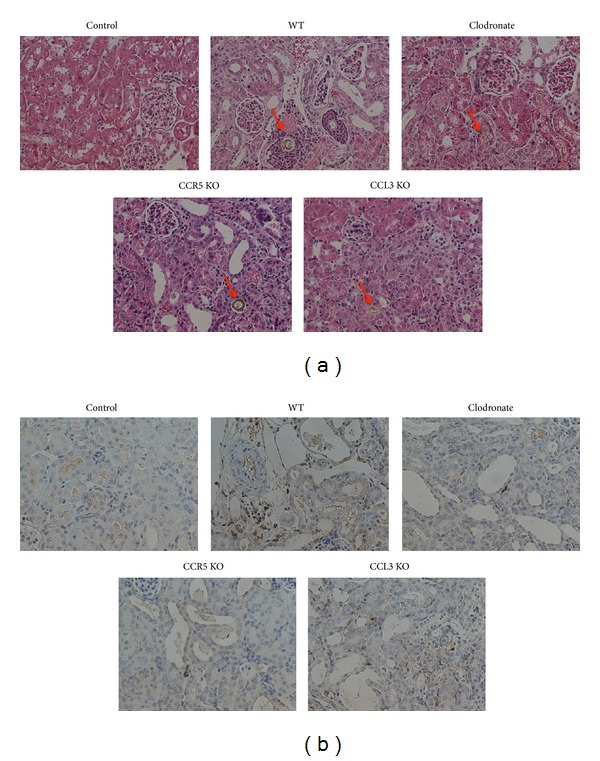
DHA crystals and macrophage localization after excessive adenine intake in WT, clodronate treated, CCR5, and CCL3 KO animals. (a) HE staining of the groups mentioned before showing the DHA crystals deposition in renal tissue, indicated by red arrows. In panel (b), IHC for CD11b is represented, showing macrophage infiltration in the different groups.

To assure that macrophages are leading actors in the onset of TIN we provided adenine-enriched food to the animals and depleted the phagocytic population using the liposome clodronate solution. As it can be observed in Figure 2(a), the excessive adenine intake leads to the formation of 2,8-dihydroxyadenine (DHA) crystals, which is not observed in control animals. An overall view of the HE stained slide shows more crystals (in number and size) in WT animals than in the other groups (data not shown). Interestingly, in WT animals there is a massive inflammatory infiltrate surrounding the crystals, a phenomenon not observed when the animals were treated with clodronate (Figure 2(a)). Moreover, when we perform an IHC assay for CD11b, we observe an increase in the staining in WT animals, quite different from control and macrophage depleted mice (Figure 2(b)).

**Figure 3:**
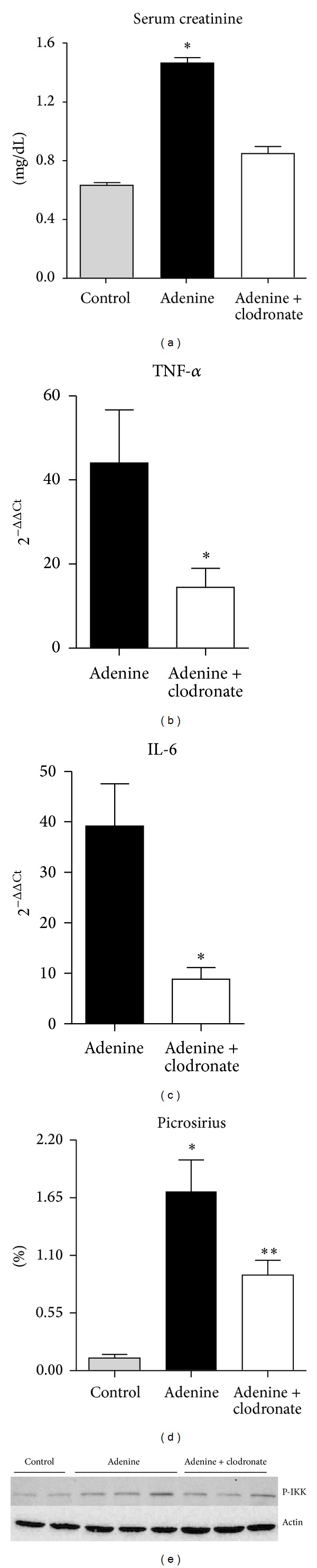
Macrophage depletion leads to protection in adenine-induced TIN. (a) Renal function of control, adenine, and adenine + clodronate mice was evaluated by serum creatinine (*P < 0.05 versus control and adenine + clodronate). The inflammatory profile from these animals was measured and indicated by the levels of TNF-*α* and IL-6 (panels (b) and (c), resp., *P < 0.05 versus adenine). Also, fibrosis was assessed by the picrosirius staining (panel (d), *P < 0.05 versus control and adenine + clodronate and **P < 0.05 versus control). (e) Protein expression of p-IKK in control, adenine, and clodronate treated animals.

Also, Figure 3(a) shows that clodronate treated mice exhibited decreased levels of serum creatinine, indicating that if macrophages are not present, the renal dysfunction is reduced. In addition, the macrophage depletion significantly abrogated the inflammatory process, as observed by attenuation of TNF-*α* and IL-6 expressions, as well as a decrease in p-IKK protein levels, a marker of NF-*κ*B activation [7] (Figures 3(b), 3(c), and 3(e)). Finally, to confirm that macrophages are important mediators of renal fibrosis, we measured collagen deposition and observed that the clodronate group has significantly less picrosirius staining than the untreated group (Figure 3(d)). Put together, these results show that after a reduction in the macrophage population there is a striking protection regarding renal function, inflammation, and fibrotic aspects.

### 3.3. CCR5 Knockout Animals Show Less Renal Dysfunction in Adenine-Induced TIN

**Figure 4:**
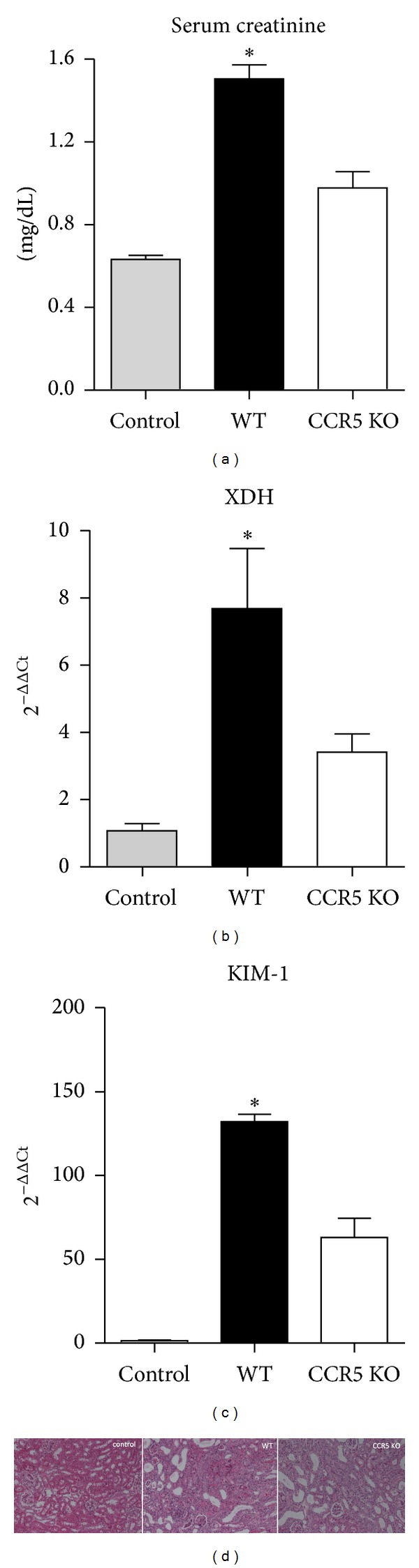
Lack of CCR5 promotes less renal dysfunction in TIN. (a) Serum creatinine levels were dosed in control (that received standard food), WT, and CCR5 animals (that received adenine-enriched food). In panels (b) and (c) we can observe the transcripts levels of XDH and KIM-1, respectively. (d) H&E staining of kidney sections of the groups described before. *P < 0.05 versus other groups.

As CCR5 is a common receptor for several macrophages recruiting chemokines, we decided to use animals with gene deletion for such molecule and provided adenine-enriched food to them. The lack of this receptor led to less inflammatory infiltration around the DHA crystals (Figure 2(a)). Also, Figure 4(a) shows that, when compared to WT animals, CCR5 knockout (KO) mice show decreased levels of serum creatinine. In addition, as observed previously [7], xanthine dehydrogenase (XDH) is an important mediator of adenine-induced TIN, as it promotes the formation of adenine crystals, leading to their deposition within the kidney. Here, we show that the KO animals also showed less gene expression of this enzyme (Figure 4(b)). Furthermore, the expression of KIM-1, a marker of kidney injury, is significantly reduced in CCR5 KO animals (Figure 4(c)). Finally, in opposition to WT animals, which show a clear alteration in renal architecture after adenine intake, the CCR5 KO group shows a better maintenance of the histological aspect (Figure 4(d)). Together, these results show that lack of CCR5 provides a better outcome during TIN.

### 3.4. Lack of CCR5 Reduces Macrophage Infiltration and Renal Inflammation

**Figure 5:**
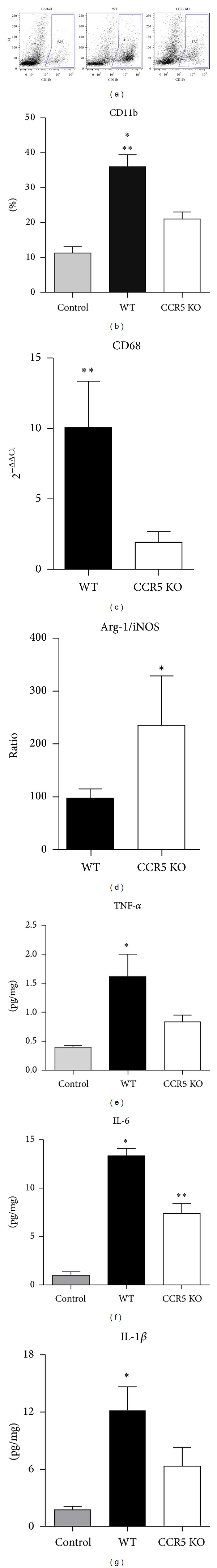
Macrophage recruitment in renal tissue is reduced in CCR5 KO animals, leading to less inflammation. (a) Representative image of the macrophage infiltration in the kidney, evaluated by CD11b positive cells. In panel (b) we can observe the quantification of the total data from panel (a) (*P < 0.01 versus Control and **P < 0.05 versus CCR5 KO group). The gene expression of CD68 and the ratio regarding the mRNA levels of arginase-1/iNOS can be checked at panels (c) and (d), respectively (**P < 0.05 versus CCR5 KO and *P < 0.05 versus WT). Also, the protein levels of the proinflammatory molecules TNF-*α*, IL-6, and IL-1*β* were evaluated in the same animals and the results are shown in panels (e), (f), and (g), respectively (*P < 0.05 versus control and CCR5 KO groups and **P < 0.05 versus control).

Once we believe that the protection previously observed occurs because of less macrophage trafficking, we decided to assess the expression of this cell population in the injured tissue. As observed in Figures 5(a) and 5(b), WT animals that received adenine-enriched food show a fourfold increase of infiltrated macrophages into the kidney. On the other hand, CCR5 KO mice exhibited an astonishing decrease of macrophages presence in the renal tissue. In accordance with this, we observed less staining for macrophages in CCR5 KO animals, when compared to WT mice (Figure 2(b)). Moreover, in the CCR5 KO group, we could also observe less gene expression of the macrophage marker CD68 (Figure 5(c)). To go further and evaluate if the macrophage population are differentially expressed, we decided to check the expression of the M1 macrophage population iNOS, as well as the M2 macrophage population arginase-1. As we can observe in Figure 5(d), there is a higher ratio of arginase-1/iNOS in the CCR5 KO group, indicating that in these animals there is a shift from a proinflammatory to an anti-inflammatory profile. A consequence of less infiltrating macrophages is a significant reduction in the renal expression of the proinflammatory molecules TNF-*α*, IL-6, and IL-1*β* and concomitant decrease of p-IKK expression (Figures 5(e)–5(g) and Figure 7(d)). Together, all these data show that lack of CCR5 attenuates macrophage infiltration with subsequent decrease of the inflammatory process.

### 3.5. CCR5 Expression Regulates Fibrosis Deposition

**Figure 6:**
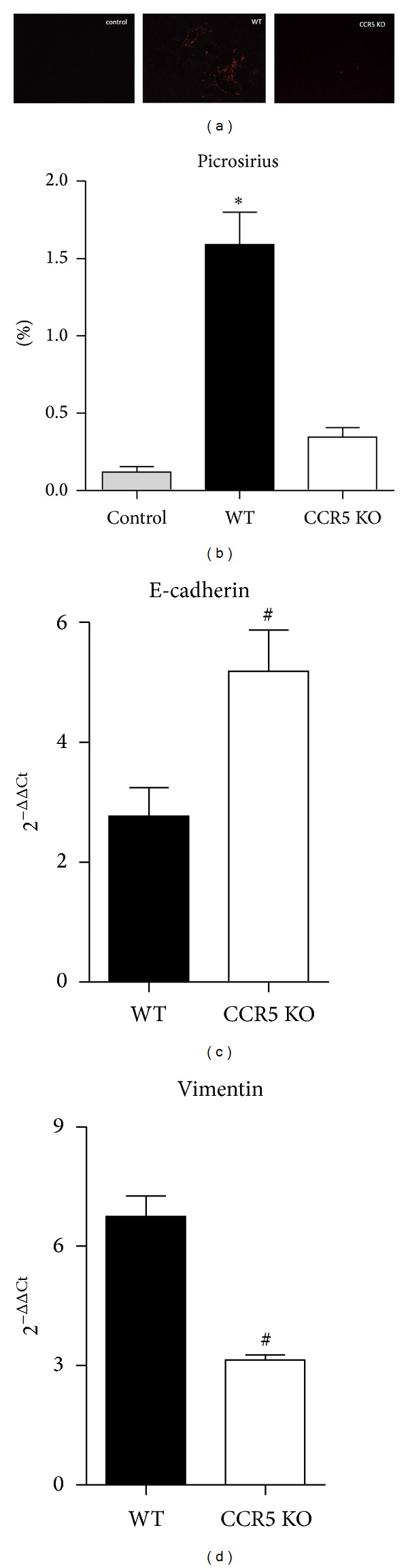
CCR5 deficient animals show less fibrosis after adenine-enriched food intake. (a) Representative image of the picrosirius staining in renal tissue of the indicated groups. (b) Graphic quantification of the picrosirius positive area of each group (*P* < 0.01 versus control and CCR5 groups). The gene levels of E-cadherin (panel (c)) and vimentin (panel (d)) were also assessed (^#^ *P* < 0.05 versus WT).

Recently, the search to avoid chronicity of acute insults is a big challenge for the medical sciences. To check if the lack of CCR5 could attenuate fibrosis deposition as a consequence of less macrophage infiltration, we evaluated some fibrotic markers. As observed in Figure 6(a), picrosirius staining (which evidences collagen deposition) showed that, in CCR5 KO animals, when compared to WT group, there is a striking reduction of such fibrotic hallmark (Figure 6(b)). In addition, Figures 6(c) and 6(d) show that CCR5 KO animals present higher level of E-cadherin (an epithelial marker) and less levels of vimentin (a mesenchymal marker), showing that the process of epithelial-to-mesenchymal transition (EMT) is reduced when there is an attenuation of macrophages recruitment. Altogether, these results suggest that since there is a reduction of macrophages recruitment signaling, the subsequent fibrosis deposition is markedly downregulated.

### 3.6. CCL3 Is an Important Mediator of TIN

**Figure 7:**
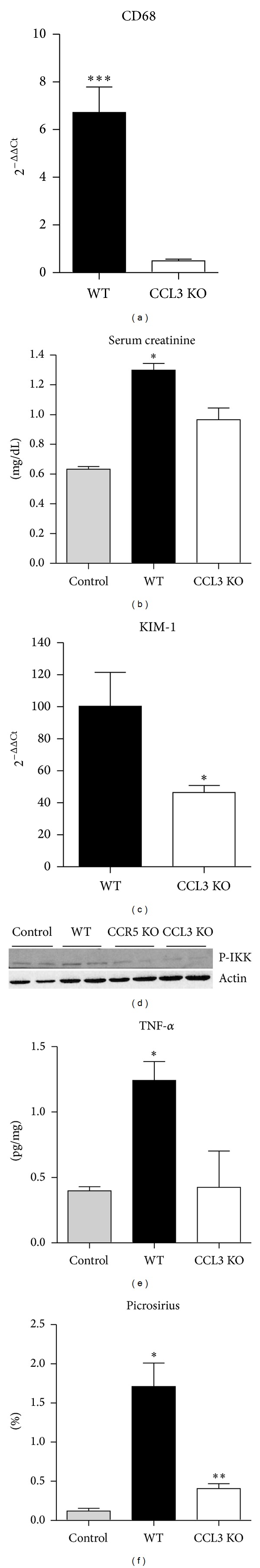
CCL3 KO animals also show a better outcome after adenine-induced TIN. (a) CD68 expression in WT and CCL3 KO animals (***P < 0.001 versus CCL3 KO group). The renal function was evaluated by serum creatinine levels (panel (b), *P < 0.05 versus control and CCL3 KO groups) and KIM-1 transcripts levels (panel (c), *P < 0.05 versus WT). (d) Western Blotting for p-IKK was performed in control, WT, and KO groups. (e) Protein levels of TNF-*α* in renal tissue (*P < 0.05 versus control and CCL3 KO groups). Fibrosis was evaluated by picrosirius staining and the graphic quantification can be observed in panel (f) (*P < 0.01 versus Control and CCL3 KO groups and **P < 0.05 versus control).

One of the chemokines that uses the CCR5 receptor to recruit macrophages to the inflammatory site is CCL3. So, to evaluate if this molecule contributes to the progression of TIN, we provided adenine-enriched food to CCL3 KO animals. We could observe that CCL3 KO animals also have less inflammatory infiltrate surrounding the DHA crystals and decreased CD11b staining in renal tissue (Figure 2). Moreover, as observed in Figure 7, the knockout mice showed less expression of the macrophage marker CD68, followed by a better renal function outcome, with decreased levels of serum creatinine (Figures 7(a) and 7(b)). Also, as stated before, the marker of renal dysfunction—KIM-1—is significantly reduced in CCL3 KO animals (Figure 7(c)). This afforded protection could also be observed regarding TNF-*α* production and p-IKK expression in the kidney tissue (Figures 7(d) and 7(e)). Consequently, there is a striking reduction in collagen deposition in the renal tissue of CCL3 KO mice, measured by picrosirius staining (Figure 7(f)). Together, these data indicate that CCL3 contributes, at least in part, to the development of nephritis, probably by increasing the recruitment of macrophages.

## 4. Discussion

It has been widely accepted that immune cells recruitment and infiltration are key elements in the onset of a number of renal diseases, including acute renal injury, glomerulonephritis, and progressive kidney injury. TIN is an important cause of kidney injury, representing almost twenty percent of acute kidney failure presentations in older adults [8], and is characterized by macrophage infiltration and decline of renal functions. Parallel events like matrix deposition occur as inflammatory process becomes more severe, mainly by fibroblasts activation at interstitial sites which triggers production of collagen and consequently fibrosis [9]. In this study, adenine-fed mice developed TIN by deposition of kidney stones. This occurs because adenine is a substrate for XDH, which leads to the formation of an insoluble compound, named 2,8-dihydroxyadenine (DHA), that precipitates in tubule-interstitial compartment causing TIN [10]. As shown, XDH gene expression rises in wild type mice, and consequently KIM-1 and serum creatinine levels evidenced tubular damage. The apoptosis of resident cells and, as a result, the tissue damage contribute to generating an inflammatory environment which apparently contributes to progression of the disease [11].

Inflammation has been shown to play an important role in the development of TIN in the adenine experimental model [7]. In this sense, it is feasible to suggest that the immune cells might be responsible, at least in part, for this injury. The involvement of macrophages infiltration in the renal tissue after an insult was already detected in different works [5, 12]. Several groups have attributed reduced morphologic kidney damage when macrophages were depleted in different experimental models [13, 14].

Based on this information, we can suggest that the protection observed in the knockout animals can be explained by the absence of activated macrophage on the tissue. Other studies using different experimental model showed reduction in intratumoral accumulation of macrophages in CCR5 deficient mice [15–17]. Also, works assigned the blockage of CCR5 on reduced leukocyte infiltration in the kidneys of antiglomerular basement membrane glomerulonephritis [18].

To the same extent, mice deficient for CCL3 had lower tubular damage, attenuated inflammatory status, and decreased collagen deposition when compared to WT littermates. This chemokine, a chemoattractant member of the CC-chemokine family, is a ligand of CCR5 [19] and has been shown to mediate the migration of neutrophils, lymphocytes, and monocytes* in vitro* and* in vivo* [20–22]. Moreover, CCL3 has been implicated in regulation of cancer cell growth and metastasis of different tumors [15, 23]. Furthermore, chemokines that engage in CCR5 are able to trigger a downstream cascade to generate chemoattractant and proinflammatory mediators, like IL-1*β*, TNF-*α*, and IL-6 [20], corroborating the decreased expression of these molecules in CCR5 and CCL3 KO mice. In this scenario, a lesion caused by DHA crystals leads to an inflammatory environment that, among a variety of stimuli, the chemokine production by resident cells seems to be crucial to inflammatory cells recruitment at injured site and, to a greater extent, macrophages. Thus, CCL3 is secreted by local cells and, later, sensitizes CCR5 positive cells (e.g., macrophages), which will be recruited to the site of injury and will also produce more of this chemokine, exacerbating this inflammatory response. Lack of CCL3 or CCR5 impairs the inflammatory event, avoiding it from reaching a disordered and chaotic environment in which the disease progresses to fibrosis and later to loss of renal function. Still, a couple of works have shown that inflammation, and specifically NF-*κ*B activation, is an important mediator of TIN induced by adenine. One of the studies showed that impairment of Nrf2 could lead to increased inflammation, while another proved that when NF-*κ*B is inhibited the progression of TIN is clearly abrogated [24, 25].

Regarding the shift from an M1 to and M2 macrophage profile in the CCR5 KO group, one can state that the presence of an inflammatory environment stimulates the differentiation of the subtype of macrophages known as M1, which has been described as important for increased production of TNF-*α*. This cytokine, besides an autocrine/paracrine effect in the macrophages themselves, also induces apoptosis/necrosis of the renal parenchyma cells, such as endothelial cells, tubular cells, and podocytes [26–28]. In turn, tissue repair mechanisms are usually activated to regulate the inflammatory process through the release of molecules such as IL-10 and TGF-*β* [29]. This microenvironment dominated by anti-inflammatory cytokines inhibits M1 macrophages and directly promotes the differentiation of monocytes into anti-inflammatory macrophages, also known as M2 [30].

Finally, macrophages participate in matrix remodeling by producing matrix metalloproteinases that degrade the basement membrane and interstitial area, facilitating the recruitment of inflammatory cells to the site of injury [31]. Moreover, to promote wound healing of tissue, they secrete profibrotic mediators like TGF-*β*1 and PDGF that promote fibroblast proliferation and activation to myofibroblasts, which are the main cells involved in maladaptive repair process by synthesizing collagen and other extracellular matrix components [17]. Also, macrophages-derived chemokines under inflammatory stress contribute to EMT of injured tubular cells, leading to a fibroblast phenotype [32]. In the absence of CCR5 or CCL3, the initial insult caused by adenine-induced TIN happens in a lesser extension because inflammatory chemokines responsible to recruit macrophages and exacerbate the events related to fibrosis is reduced.

## 5. Conclusion

In conclusion, we were able to show an important role of macrophages in TIN pathogenesis, exacerbating the insult and promoting fibrosis. In this scenario, macrophages through CCR5 and CCl3 are key elements to orchestrate inflammatory event. Anyway, a better understanding of their specific role should be clearly elucidated in future studies.
